# Association between Plasma Ascorbic Acid Levels and Postoperative Delirium in Older Patients Undergoing Cardiovascular Surgery: A Prospective Observational Study

**DOI:** 10.3390/jcdd10070293

**Published:** 2023-07-10

**Authors:** Yusuke Iizuka, Koichi Yoshinaga, Kyosuke Takahashi, Sayaka Oki, Yoshihiko Chiba, Masamitsu Sanui, Naoyuki Kimura, Atsushi Yamaguchi

**Affiliations:** 1 Department of Anesthesiology and Critical Care Medicine, Saitama Medical Center, Jichi Medical University, Saitama 330-8503, Japan; 2 Department of Cardiovascular Surgery, Saitama Medical Center, Jichi Medical University, Saitama 330-8503, Japan

**Keywords:** ascorbic acid, cardiovascular surgery, postoperative delirium

## Abstract

Background: The incidence of delirium is high in older patients undergoing cardiovascular surgery with cardiopulmonary bypass (CPB). Intraoperative tissue hypoperfusion and re-reperfusion injury, which generate reactive oxygen species (ROS), are suggested to induce delirium. Ascorbic acid is an excellent antioxidant and may reduce organ damage by inhibiting the production of ROS. This prospective observational study aimed to measure pre- and postoperative plasma ascorbic acid levels and examine their association with delirium. Methods: Patients older than 70 years of age scheduled for elective cardiovascular surgery using CPB were enrolled. From September 2020 to December 2021, we enrolled 100 patients, and the data of 98 patients were analyzed. Results: In total, 31 patients developed delirium, while 67 did not. Preoperative plasma ascorbic acid levels did not differ between the non-delirium and delirium groups (6.0 ± 2.2 vs. 5.5 ± 2.4 µg/mL, *p* = 0.3). Postoperative plasma ascorbic acid levels were significantly different between the groups (2.8 [2.3–3.5] vs. 2.3 [1.6–3.3] µg/mL, *p* = 0.037). Conclusions: In patients who undergo cardiovascular surgery with CPB, lower postoperative plasma ascorbic acid levels may be associated with the development of delirium.

## 1. Introduction

Postoperative delirium is a serious prognosis-affecting complication. Risk factors for delirium have been classified into predisposing and precipitating factors. Common predisposing factors are male sex, older age, dementia, and functional disabilities. Among precipitating factors, drugs (especially sedative-hypnotic and anticholinergic agents), surgery, anesthesia, high pain levels, anemia, infections, acute illness, and acute exacerbation of chronic illness are the most reported [1]. In cardiac surgery, using cardiopulmonary bypass (CPB) with continuous flow, hypothermia, microembolization, impairment of cerebral oxygenation, and autoregulation that coincides with heart dysfunction further increases the risk of postoperative delirium. Therefore, the incidence of postoperative delirium is high in patients undergoing cardiovascular surgery [2]. Postoperative delirium requires early diagnosis and treatment because it causes acute cognitive disorder, prolonged mechanical ventilation, prolonged length of hospital stay, high hospital mortality, and cognitive decline [3,4]. However, as no established prophylactic drugs exist, only nonpharmacological treatments are employed [5]. Older patients undergoing cardiovascular surgery are a particularly high-risk group for developing postoperative delirium, so delirium-preventive drugs are needed.

Ascorbic acid (vitamin C) has excellent antioxidant properties and is thought to reduce organ damage by inhibiting the production of reactive oxygen species (ROS) generated by tissue hypoperfusion and re-reperfusion injury [6]. Several studies used ascorbic acid in cardiac surgery to prevent the new onset of organ damage. In particular, administering ascorbic acid significantly reduced the new onset of postoperative atrial fibrillation [7]. Although the mechanism of the development of postoperative atrial fibrillation is not completely understood, the inflammation and subsequently developed oxidative stress caused by ischemic and traumatic injury play the main role [8]. Ascorbic acid, an antioxidant and free radical scavenger, possibly decreases oxidative stress and prevents postoperative atrial fibrillation. Postoperative delirium may also be considered brain damage that occurs through a similar mechanism; ascorbic acid might prevent postoperative delirium. However, there are no studies on the association between ascorbic acid administration and the development of delirium in patients undergoing cardiovascular surgery.

We hypothesized that administering ascorbic acid could reduce the incidence of delirium in older patients scheduled to undergo cardiovascular surgery with CPB. We conducted a prospective observational study to measure pre- and postoperative plasma ascorbic acid levels and examine their association with the development of postoperative delirium in older patients who underwent cardiovascular surgery using CPB. If low preoperative or postoperative plasma ascorbic acid levels are associated with developing postoperative delirium, the hypothesis that ascorbic acid can prevent postoperative delirium may be supported. The results of this study will help in deciding when ascorbic acid supplementation should be given to patients in subsequent studies that will examine the effect of the actual administration of ascorbic acid.

## 2. Materials and Methods

This single-center, prospective, observational study was approved by the Research Ethics Committee of Saitama Medical Center Jichi Medical University (S21-131). This study was conducted in accordance with the Declaration of Helsinki and registered with the University Hospital Medical Information Network (UMIN000042104). The study investigated whether pre- and postoperative plasma ascorbic acid levels are associated with postoperative delirium in older patients who underwent cardiovascular surgery using CPB. The inclusion criteria required patients to be older than 70 years of age and scheduled for elective cardiovascular surgery using CPB. Patients with cognitive disorders, those who could not understand the details of this study, or those requiring urgent surgery were excluded. Written informed consent was obtained from all patients.

### 2.1. Perioperative Management

All patients were fitted with an arterial catheter placed in the radial artery. Before or immediately after induction of anesthesia, 5 mL of blood was drawn from the arterial catheter to examine the preoperative plasma ascorbic acid levels. Anesthetic management was similar in all the patients. Anesthesia was induced by intravenous administration of midazolam (or propofol), fentanyl, remifentanil, and rocuronium. Anesthesia was maintained with sevoflurane, except during CPB. During CPB, propofol was used to maintain anesthesia. Mild hypothermia (bottom temperature set to 30 °C) was used in valvular surgery, and deep hypothermia (bottom temperature set to 25 °C) was used in aortic surgery requiring circulatory arrest with CPB. All patients were transferred to the intensive care unit (ICU) after surgery and extubated after hemodynamic stabilization. Postoperative plasma ascorbic acid levels were measured from plasma samples drawn 24 h after the first drawn preoperative sample (usually in the morning on the postoperative day 1). An ICU nurse evaluated postoperative delirium until ICU discharge using the Confusion Assessment Method for the Intensive Care Unit (CAM-ICU). The CAM-ICU was checked routinely 2–3 times daily for all patients.

### 2.2. Statistical Analysis

No study has investigated the association between plasma ascorbic acid levels and postoperative delirium; therefore, we cannot estimate an adequate sample size. We collected data from 100 older patients scheduled for cardiac surgery with CPB.

Shapiro–Wilk tests were used to assess the normality of continuous variables. Normally distributed variables were expressed as mean ± standard deviation (mean ± SD), while non-normally distributed variables were expressed as medians (interquartile range [IQR]). Where appropriate, continuous variables were compared between groups using the two-sample t-test and Mann–Whitney U test. Categorical variables were expressed as numbers. Where appropriate, Fisher’s exact or chi-square test was used to compare categorical variables. After univariate analysis, multiple logistic regression analysis was used to adjust for the possible confounding factors associated with delirium (plasma level of ascorbic acid, intubation time, and length of ICU stays referred from a systematic review of risk factors for delirium in cardiac surgery) [9]. The area under the receiver-operating characteristic curves (AUROC) for the plasma ascorbic acid ability to predict the development of postoperative delirium was calculated and compared using the method described by DeLong et al. [10]. Optimal cutoff values were calculated by maximizing the Youden index. Pearson’s correlation coefficient was used to evaluate the relationship between plasma ascorbic acid and the duration of postoperative delirium. *p* < 0.05 was considered statistically significant. All statistical analyses were performedwith EZR (Saitama Medical Center, Jichi Medical University, Saitama, Japan), a graphical user interface for R (version 3.6.3) (R Foundation for Statistical Computing, Vienna, Austria). More precisely, EZR is a modified version of R commander (version 2.6-2) designed to add statistical functions frequently used in biostatistics. [11].

## 3. Results

**Figure 1:**
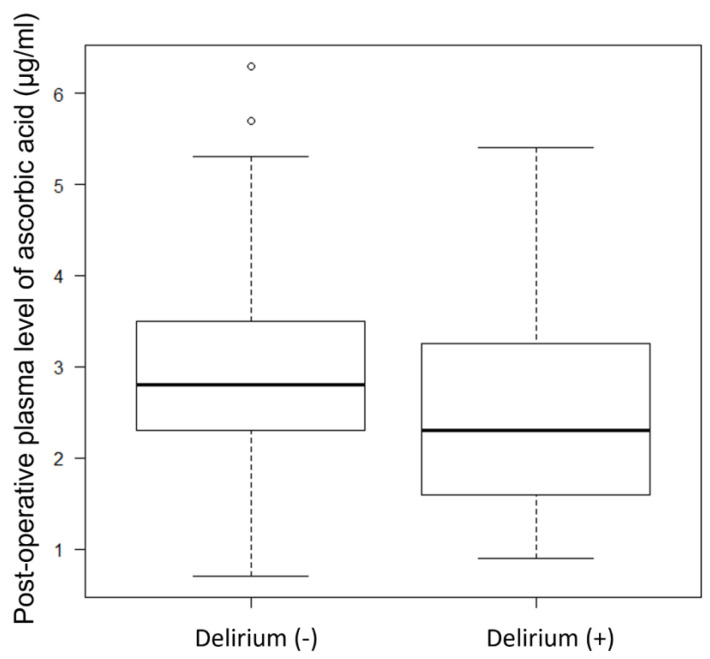
Box plot of postoperative plasma ascorbic acid levels.

From September 2020 to December 2021, we enrolled 100 patients. However, postoperative plasma ascorbic acid levels were not measured in two patients. Therefore, the data of 98 patients were used in our analysis. Table 1 shows the characteristics of the enrolled patients. In total, 31 patients developed delirium (31.6%), while 67 did not. In the delirium group, the duration of delirium (determined as the time from CAM-ICU positive to CAM-ICU negative or ICU discharge, whichever came first) was 16.6 (11.6–41.3) h. Preoperative plasma ascorbic acid levels did not differ between the non-delirium and delirium groups (6.0 ± 2.2 vs. 5.5 ± 2.4 µg/mL, *p* = 0.3). Postoperative plasma ascorbic acid levels differed significantly (2.8 [2.3–3.5] vs. 2.3 [1.6–3.3] µg/mL, *p* = 0.037) between the groups. Figure 1 depicts a box plot of each group’s postoperative plasma ascorbic acid levels.

The other factors differed significantly between the groups, including the variation of procedures, the European system for cardiac operative risk evaluation score (EuroSCORE II), CPB time, operation time, and intubation time in the ICU.

Table 2 shows the results of the multivariate analysis generated using data on postoperative ascorbic acid levels, intubation time in the ICU, and length of ICU stay to adjust for possible confounding factors associated with developing delirium. The postoperative plasma ascorbic acid levels were found to be associated with delirium (odds ratio, 0.576; 95% confidence interval: 0.352–0.941; *p* = 0.028). The generated AUROC for the ability of postoperative plasma ascorbic acid to predict the development of postoperative delirium showed a low predictability (AUROC 0.632, 95% confidence interval, 0.506–0.758). The best cutoff value in the plasma level of postoperative ascorbic acid was 2.5 µg/mL (Sensitivity 0.677, Specificity 0.612).

## 4. Discussion

This study revealed that a decrease in the postoperative plasma ascorbic acid levels, but not the preoperative ascorbic acid levels, may be associated with the development of delirium. To our knowledge, this is the first study to precisely investigate the association between plasma ascorbic acid levels and the development of delirium in older patients scheduled to undergo cardiovascular surgery with CPB.

In cardiac surgery with CPB, altered perfusion during CPB may cause hypoperfusion, hypoxemia, microemboli, and a systemic inflammatory response [12,13]. Those factors cause ischemia/reperfusion injury, which has a pathophysiological pathway comprising overwhelming amounts of ROS, causing endothelial dysfunction, cellular injury, and multiple organ failure.

Ascorbic acid is an essential water-soluble vitamin. It is an electron donor that directly scavenges ROS. Moreover, ascorbic acid prevents the generation of new ROS through its suppressive effects on the nicotinamide adenine dinucleotide phosphate (NADPH) oxidase pathway and assists in the recycling of other antioxidants [14]. The antioxidant effect of ascorbic acid reduces endothelial permeability. Ascorbic acid levels decrease after cardiac surgery due to the generation of ROS [15,16]. Therefore, low plasma ascorbic acid levels may be due to the generation of substantial amounts of ROS. They may also be associated with multiple organ damage, and in such a situation, the supplementation of depleted ascorbic acid may minimize the effects of ROS.

Several studies reported the effects of supplementation with ascorbic acid in patients who underwent cardiac surgery. Uzun et al. reported that ascorbic acid significantly enhanced endothelium-dependent vasodilatation in the radial circulation of patients with coronary artery disease [17]. This may be attributed to an enhancement in the synthesis or prevention of the breakdown of nitric oxide. These results showed that ascorbic acid improved defective endothelial function. In a pragmatic study, the most studied area of ascorbic administration and organ damage in cardiovascular surgery is its effect in preventing postoperative atrial fibrillation. Many studies and meta-analyses have shown that ascorbic acid administration may reduce postoperative atrial fibrillation [7,18]. Other studies reported a beneficial effect of administering ascorbic acid in shortening the duration of mechanical ventilation, length of stay in the ICU, and length of stay in the hospital [5]. Ascorbic acid may improve organ functions after cardiac surgery, although the protective effects against cerebral ischemic events, postoperative cognitive dysfunction, or delirium were unknown.

This study focused on delirium as postoperative brain damage. Brain tissue is susceptible to oxidative damage due to its high content of polyunsaturated fatty acids and high oxygen demand. Ascorbic acid is the most important antioxidant in the brain. Ascorbic acid levels are much higher in brain cells (2–10 mM) and are up to four times higher in the cerebrospinal fluid (200–400 µM) compared to that in the plasma (40–60 µM), owing to its active transport via the sodium-dependent vitamin C transporter-2 (SVCT2) transporter [19]. In a cat model, brain vitamin C levels declined markedly during cerebral ischemia [20]. In ascorbic acid-rich organs, such as the brain, we considered that changes in ascorbic acid levels in the brain tissue might be strongly associated with postoperative neurological complications such as delirium.

Few studies have investigated the association between plasma concentrations of ascorbic acid or the administration of ascorbic acid and postoperative delirium. Hill et al. studied the association between perioperative plasma levels of ascorbic acid and vitamin E and inflammatory reaction, oxidative stress, organ dysfunction, and clinical outcomes in 34 patients who underwent cardiac surgery with CPB [21]. Their analysis showed no statistically significant association of ascorbic acid with inflammation, oxidative stress, or organ dysfunction levels in patients with preoperative suboptimal ascorbic acid status (<9 µg/mL) or in patients with a perioperative decrease of >50% ascorbic acid after surgery. Postoperative delirium was one of the outcomes they reported that was not associated with perioperative plasma levels of ascorbic acid. However, they did not evaluate the association between the nadir of plasma ascorbic acid and delirium, and the definition of delirium diagnosis was unclear. Moreover, only five patients had developed postoperative delirium, with an incidence rate of 15%. The incidence rate they reported was much lower than that reported in our results, although this difference might be explained by the younger age of the patients (median age of 69–72). Their study’s sample size was too small to allow any conclusions. Another study investigated the association between delirium-free days and the administration of ascorbic acid in patients in septic shock. Park et al. reported that in a retrospective study using propensity score matching, ascorbic acid and thiamine administration showed no association with delirium-free days among patients in septic shock [22]. Sepsis-associated delirium is one of the symptoms of sepsis-associated encephalopathy, so the etiology of developing delirium was more complex than postoperative delirium [23]. Meta-analysis of several randomized controlled studies of a high quality showed that administering ascorbic acid could not reduce organ dysfunction or mortality in patients with sepsis [24]. Ascorbic acid as an antioxidant and free radical scavenger might suit non-septic patients such as elective postoperative patients. The literature review revealed little about the relationship between delirium and ascorbic acid.

Our results showed that the plasma level of ascorbic acid declined by over 50% from the preoperative to the postoperative value. They revealed that lower postoperative plasma ascorbic acid levels, not preoperative plasma levels, were associated with the incidence of postoperative delirium. As hypothesized, CPB induced ischemia/reperfusion injury and generated ROS. Substantial ascorbic acid was consumed to scavenge ROS. The lower postoperative ascorbic acid plasma levels might be the result of severe ischemia/reperfusion injury and might be the cause of postoperative delirium. However, if the CPB consumes ascorbic acid, it should not be surprising that those with low preoperative ascorbic acid levels would develop delirium. A possible explanation was that the nadir in the plasma or brain level of ascorbic acid might be associated with postoperative delirium. However, the ability to predict postoperative plasma levels of ascorbic acid was low. A decrease in the ascorbic acid levels in the brain of patients who developed delirium might be more drastic, and a threshold level of ascorbic acid in the brain tissue may be associated with developing delirium. However, we are unable to speculate about changes in ascorbic acid levels in the brain tissue due to the nature of this study. Our results imply that maintaining the plasma ascorbic acid level may prevent delirium, which supports the hypothesis that administering ascorbic acid could reduce the incidence of postoperative delirium. The peak level after a single dose of intravenous ascorbic acid was reached within 1 h and returned to normal in 24 h in a volunteer study [25]. This pharmacological characteristic means that the optimal timing of ascorbic acid administration may be preoperatively or at least 1 h before the start of CPB.

The beneficial effect of ascorbic acid in protecting the brain from damage after cardiac surgery is unknown. However, ascorbic acid has been shown to effectively reduce the size of cerebral infarction in animal models [26,27]. Further studies are needed to investigate the beneficial effects of ascorbic acid in reducing the incidence of postoperative delirium. We have started a pilot study to investigate the effect of ascorbic acid supplementation at 2 g/day for 2 days on the incidence rate of postoperative delirium in older patients who underwent cardiac surgery with CPB (Registration number: jRCTs031220690).

Our study had several limitations. First, we could not adjust for other residual confounding factors associated with the incidence of delirium, owing to the small sample size. We only used three variables in the multivariate analysis because 10 events per variable are generally allowed in logistic regression. We chose the intubation time and length of ICU stay from our variables, in addition to the postoperative plasma ascorbic acid level, as possible confounders for the multivariate analysis. Variables were referred to from a systematic review of risk factors for delirium in cardiac surgery that identified eight risk factors (aging, diabetes, preoperative depression, mild cognitive impairment, carotid artery stenosis, NYHA functional class III or IV, time of mechanical ventilation, and length of ICU stay) [9]. We did not collect data about preoperative depression, mild cognitive impairment, and carotid artery stenosis. Aging might not be a suitable confounding factor, as we already chose older patients. From the other identified risk factors, we chose mechanical ventilation and the length of ICU stays because the *p*-values of those variables were <0.15 in the univariate analysis. The variety of surgery might be an important residual confounding factor. In our patients, those who underwent aortic reconstruction (n = 32, patients with aortic surgery and part of composite surgery) had a higher incidence of postoperative delirium than those who did not (incidence rate of delirium 53% (17/32) and 21% (14/66), respectively). Aortic reconstruction may lead to a higher risk of developing postoperative delirium. Our results may have differed if we had a much larger sample size, large enough to use other pre- and intraoperative variables to generate a multivariate analysis. Second, we could not evaluate the severity of delirium. The lower plasma or brain level of ascorbic acid would correlate with the development of severer delirium or a longer duration of delirium. However, we could not find any correlation between the postoperative plasma level of ascorbic acid and the duration of delirium (r = 0.05 95% confidence interval, −0.149–0.247). Third, we did not collect data on subsyndromal delirium. Subsyndromal delirium is characterized by a milder cognitive dysfunction and does not meet the formal criteria for delirium; its symptoms are highly varied [28]. Some patients in our study did not develop postoperative delirium but had hallucinations. Investigating the association between perioperative plasma levels of ascorbic acid and subsyndromal delirium in patients who did not develop delirium might produce new findings. With regards to subsyndromal delirium, further research will be needed. Although this study had many limitations, our results have clinical importance by presenting basic demographic data on the association between plasma ascorbic acid levels and the incidence of postoperative delirium.

## 5. Conclusions

In patients who underwent cardiovascular surgery with CPB, lower postoperative, not preoperative, plasma levels of ascorbic acid were associated with developing delirium. Further studies are needed to investigate the association between ascorbic acid and postoperative delirium.

## Figures and Tables

**Table 1:** Patient characteristics.

	Delirium (−)	Delirium (+)	*p*-Value
N	67	31	
Age (years)	76 (73–78)	77 (74–79)	0.36
Sex (male, female)	38, 29	20, 11	0.47
Hypertension	54	25	1
Diabetes mellitus	10	4	0.8
Dyslipidemia	32	15	0.96
NYHA functional class III or IV	7	2	0.53
Procedure			0.005
Valve surgery	30	4	
Aortic surgery	9	10	
Coronary artery bypass grafting	0	1	
Composite surgery	25	15	
Others	3	1	
EuroSCORE II	2.84 (1.51–5.70)	4.14 (2.89–6.97)	0.03
Preoperative plasma level of ascorbic acid (µg/mL)	6.0 ± 2.2	5.5 ± 2.4	0.30
Postoperative plasma level of ascorbic acid (µg/mL)	2.8 (2.3–3.5)	2.3 (1.6–3.3)	0.037
Rate of decline between pre- and post-plasma level of ascorbic acid (%)	50.8 (40.6–59.2)	52.8 (44.4–61.3)	0.48
CPB time (min)	120 (99–148)	160 (116–201)	<0.001
Operation time (min)	254 (222–328)	351 (267–411)	0.001
APACHE II score at ICU admission	17 (11–20)	17 (12–19)	1
APACHE III score at ICU admission	62 ± 14	62 ± 13	0.81
Intubation time (h)	10.1 (8.1–16.1)	16.1 (9.9–52.1)	0.004
Length of ICU stay (h)	95 (69–119)	108 (70–163)	0.14
Duration of delirium (h)	-	16.6 (11.6–41.3)	-

Values are shown as median (percentile 25–75) and mean ± standard or number. Abbreviations: EuroSCORE—European system for cardiac operative risk evaluation score; CPB—cardiopulmonary bypass; APACHE—Acute Physiology and Chronic Health Evaluation; ICU—intensive care unit.

**Table 2:** Results of multivariate analysis to adjust for possible confounding factors associated with delirium.

	Odds Ratio	95% CI	*p*-Value
Postoperative plasma ascorbic acid level (µg/mL)	0.576	0.352–0.941	0.028
Intubation time (h)	1.040	1.010–1.080	0.012
Length of ICU stay (h)	1.000	0.987–1.010	0.97

Abbreviations: CI—confidence interval; ICU—intensive care unit.

## Data Availability

The datasets used and/or analyzed during the current study are available from the corresponding author upon reasonable request.
